# Characterization and inhibition of inflammasome responses in severe and non-severe asthma

**DOI:** 10.1186/s12931-023-02603-2

**Published:** 2023-12-04

**Authors:** Jay C. Horvat, Richard Y. Kim, Natasha Weaver, Christopher Augood, Alexandra C. Brown, Chantal Donovan, Pierrick Dupre, Lakshitha Gunawardhana, Jemma R. Mayall, Nicole G. Hansbro, Avril A. B. Robertson, Luke A. J. O’Neill, Matthew A. Cooper, Elizabeth G. Holliday, Philip M. Hansbro, Peter G. Gibson

**Affiliations:** 1 University of Newcastle and Hunter Medical Research Institute, Newcastle, Australia; 2 University of Technology Sydney, Faculty of Science, School of Life Sciences, Sydney, Australia; 3 Centenary Institute, Centre for Inflammation, and University of Technology Sydney, Faculty of Science, School of Life Sciences, Sydney, Australia; 4 University of Montpellier, Montpellier Cancer Research Institute (IRCM), Montpellier, France; 5 The University of Queensland, School of Chemistry and Molecular Biosciences, Brisbane, Australia; 6 Trinity College Dublin, Trinity Biomedical Sciences Institute, School of Biochemistry and Immunology, Dublin, Ireland; 7 Sitala Bio Ltd., Cambridge, UK

**Keywords:** Asthma, Severe asthma, NLRP3 inflammasome, IL-1β, Inflammasome inhibition

## Abstract

**Background:**

Increased airway NLRP3 inflammasome-mediated IL-1β responses may underpin severe neutrophilic asthma. However, whether increased inflammasome activation is unique to severe asthma, is a common feature of immune cells in all inflammatory types of severe asthma, and whether inflammasome activation can be therapeutically targeted in patients, remains unknown.

**Objective:**

To investigate the activation and inhibition of inflammasome-mediated IL-1β responses in immune cells from patients with asthma.

**Methods:**

Peripheral blood mononuclear cells (PBMCs) were isolated from patients with non-severe (*n* = 59) and severe (*n* = 36 stable, *n* = 17 exacerbating) asthma and healthy subjects (*n* = 39). PBMCs were stimulated with nigericin or lipopolysaccharide (LPS) alone, or in combination (LPS + nigericin), with or without the NLRP3 inhibitor MCC950, and the effects on IL-1β release were assessed.

**Results:**

PBMCs from patients with non-severe or severe asthma produced more IL-1β in response to nigericin than those from healthy subjects. PBMCs from patients with severe asthma released more IL-1β in response to LPS + nigericin than those from non-severe asthma. Inflammasome-induced IL-1β release from PBMCs from patients with severe asthma was not increased during exacerbation compared to when stable. Inflammasome-induced IL-1β release was not different between male and female, or obese and non-obese patients and correlated with eosinophil and neutrophil numbers in the airways. MCC950 effectively suppressed LPS-, nigericin-, and LPS + nigericin-induced IL-1β release from PBMCs from all groups.

**Conclusion:**

An increased ability for inflammasome priming and/or activation is a common feature of systemic immune cells in both severe and non-severe asthma, highlighting inflammasome inhibition as a universal therapy for different subtypes of disease.

**Supplementary Information:**

The online version contains supplementary material available at 10.1186/s12931-023-02603-2.

## Key messages

- We show systemic immune cells from patients with severe and non-severe asthma have increased sensitivity to NLRP3 inflammasome activation and IL-1β release, with responses correlating with neutrophilic and eosinophilic inflammation.
- Increased inflammasome-mediated IL-1β release in systemic immune cells is similar in male *versus* female, and obese *versus* non-obese, patients, and can be readily suppressed using NLRP3 inflammasome-specific inhibitors, highlighting increased inflammasome responses as a potential therapeutic target.

**Supplementary Information:**

The online version contains supplementary material available at 10.1186/s12931-023-02603-2.

## Introduction

Severe asthma is characterized by persistent disease activity despite therapy [1, 2]. This results in increased morbidity, decreased quality-of-life, and absenteeism from school/work, with > 50% of people with severe asthma unable to maintain full-time employment [3]. In addition, patients with severe asthma account for > 50% of all asthma-associated health care costs, despite only 5–25% of patients having severe asthma (reviewed in [4–11]). Improved understanding of the immunopathological and pathophysiological processes that underpin severe asthma may provide effective therapeutic targets and/or management strategies to reduce the burden of this severe, debilitating disease.

Much clinical and experimental evidence shows that severe asthma is a heterogeneous disease, which complicates the development and application of effective therapies. Approximately 50% of patients with severe asthma have eosinophil-dominated airway inflammation and elevated type 2 (T2) responses (*e.g.* IL-4, IL-5, IL-13, immunoglobulin [Ig]E levels). However, T2-low subtypes of severe asthma also occur where patients have lower FeNO and airway and systemic eosinophils, and present with increased T1 and/or T17 responses associated with neutrophil-dominant airway inflammatory responses [6, 9, 10, 12]. Additionally, severe asthma in obese women is a distinct severe immunological clinical phenotype [6, 13–16]. Recently, T2-directed biologics were introduced as effective therapies for severe T2 asthma [17, 18]. However, alternative approaches and immunological targets are still urgently needed for severe T2-low disease [17, 18].

Accumulating clinical and experimental evidence strongly implicate excessive NLRP3 inflammasome activation and IL-1β production in severe asthma pathogenesis, particularly, neutrophil-high, T2-low subtypes [19–26]. We showed that NLRP3 inflammasome and IL-1β responses are increased in experimental models of severe, neutrophilic asthma [27]. Increased NLRP3 and IL-1β responses correlate with increased neutrophil numbers, severity of airflow obstruction, and reduced asthma control in patients, the majority of which were on ICS maintenance therapy. Most importantly, we showed that therapeutically targeting NLRP3 inflammasome responses with the highly NLRP3-specific inhibitor, MCC950, reduces IL-1β production, steroid-insensitive neutrophilic inflammation, and AHR in experimental disease. These findings demonstrate roles for inflammasome-dependent, IL-1β responses in the pathogenesis of severe, neutrophilic asthma and that increased NLRP3 activation in severe asthma may be therapeutically targeted to treat severe, T2-low disease. Whilst we showed that NLRP3 expression in sputum is associated with features of severe neutrophilic asthma, whether increased ability for NLRP3 inflammasome priming and activation and IL-1β release are key features of immune cells in other phenotypes of severe or non-severe asthma, and whether inflammasome responses in immune cells from patients with asthma can be inhibited with MCC950, is unknown.

We hypothesized that the ability for NLRP3 inflammasome priming and/or activation and IL-1β release are increased, and can be therapeutically inhibited, in immune cells from patients with severe asthma. In this study, we characterized the ability for inflammasome priming, activation, and IL-1β release from immune cells from patients with severe and non-severe asthma, and healthy controls, and examined the effects of therapeutic suppression with MCC950.

## Materials and methods

Full details are provided in the Online Repository.

### Study approvals

All procedures were performed with approval from the Hunter Area Health Service (2019/ETH01030) and University of Newcastle Human Research Ethics Committee (H-2017-

0088). All participants gave written informed consent before their inclusion.

### Study population

The study sample comprised 151 adult participants (≥ 18 years) divided into 4 diagnostic subgroups: 39 healthy controls, 59 patients with non-severe (mild-moderate, stable) asthma, 36 patients with severe stable and 17 with severe exacerbating asthma (Table 1). Details on participant recruitment, asthma diagnosis, stratification into severe and non-severe (stable or exacerbating), and eosinophilic and non-eosinophilic, asthma and obese and non-obese participants and exclusion criteria are outlined in the Supplementary Materials. Sputum samples were induced and processed as previously described [28].

**Table 1:** Subject characteristics

		Asthma status					Pairwise comparisons			
Characteristic	Class or Statistic	Healthy (*N* = 39)	Non severe (NSA; *N* = 59)	Severe (SA[S]; *N* = 36)	Severe exacerbating (SA[E]; *N* = 17)	Overall *P* value	NSA vs Healthy	SA(S) vs Healthy	SA(S) vs NSA	SA(E) vs SA(S)
Age (years)	Mean (SD)	45.0 (17.6)	52.1 (17.2)	62.9 (14.1)	58.0 (18.5)	< 0.001	0.042	< 0.001	0.003	0.318
Sex	Female	28 (72%)	42 (71%)	17 (47%)	11 (65%)	0.079	0.948	0.030	0.020	0.234
	Male	11 (28%)	17 (28%)	19 (53%)	6 (35%)					
Race	White	38 (97%)	50 (85%)	34 (94%)	17 (100%)	0.089	0.048	0.470	0.197	1.000
	Other	1 (2.6%)	9 (15%)	2 (5.6%)						
BMI, kg/m2	Mean (SD)	28.5 (5.8)	29.9 (6.3)	29.7 (7.8)	30.6 (8.4)	0.668	0.257	0.444	0.877	0.696
Obesity status	Non-obese	16 (41%)	13 (22%)	10 (28%)	6 (35%)	0.079	0.044	0.228	0.526	0.578
	Obese	23 (59%)	46 (78%)	26 (72%)	11 (65%)					
Asthma characteristics										
Age of symptom onset, years	Median (Q1, Q3)	N/A	12.0 (4.0, 27.5)	7.0 (3.5, 35.0)	11.0 (5.0, 28.5)	0.789	N/A	N/A	0.657	0.454
	Mean (SD)	N/A	17.1 (16.5)	18.9 (22.0)	20.4 (20.9)	0.792	N/A	N/A	0.655	0.804
Mean ACQ-6	Median (Q1, Q3)	N/A	0.67 (0.33, 1.17)	1.17 (0.42, 1.84)	2.50 (1.17, 3.50)	0.001	N/A	N/A	0.036	0.007
	Mean (SD)	N/A	0.89 (0.81)	1.30 (1.02)	2.28 (1.48)	< 0.001	N/A	N/A	0.054	0.023
Asthma control (*N*, %) (ACQ-6 classification)	> 1.50 0.75–1.50 < 0.75	N/A	10 (17%) 18 (31%) 30 (52%)	12 (33%) 10 (28%) 14 (39%)	11 (65%) 2 (12%) 4 (24%)	0.005	N/A	N/A	0.179	0.094
Exacerbations, past 12 months										
Number of ED visits (*N*, %)	0 1 ≥2	N/A	53 (93%) 3 (5.3%) 1 (1.8%)	33 (94%) 1 (2.9%) 1 (2.9%)	13 (76%) 2 (12%) 2 (12%)	0.117	N/A	N/A	1.000	0.103
Number of hospitalizations (*N*, %)	0 1 ≥2	N/A	53 (93%) 3 (5.3%) 1 (1.8%)	33 (94%) 2 (5.7%)	14 (82%) 1 (5.9%) 2 (12%)	0.248	N/A	N/A	1.000	0.131
Number of unscheduled Dr visits (*N*, %)	0 1 ≥ 2	N/A	39 (68%) 10 (18%) 8 (14%)	24 (69%) 4 (11%) 7 (20%)	4 (24%) 3 (18%) 10 (59%)	0.002	N/A	N/A	0.650	0.004
Number of OCS courses (*N*, %)	0 1 ≥2	N/A	37 (65%) 12 (21%) 8 (14%)	14 (40%) 4 (11%) 17 (49%)	2 (12%) 3 (18%) 12 (71%)	< 0.001	N/A	N/A	0.002	0.117
Lung function										
FEV_1_preb2pp	Mean (SD)	98.95 (12.04)	82.90 (14.79)	66.76 (19.03)	67.57 (17.75)	< 0.001	< 0.001	< 0.001	< 0.001	0.886
FVCpreb2pp	Mean (SD)	100.95 (12.55)	91.89 (14.47)	81.45 (16.27)	78.84 (15.14)	< 0.001	0.002	< 0.001	0.002	0.591
FEV_1_/FVC, %	Mean (SD)	77.51 (14.90)	72.32 (9.85)	63.54 (12.72)	67.55 (10.29)	< 0.001	0.042	< 0.001	< 0.001	0.274
Sputum inflammatory markers										
Sputum eosinophils, %	Mean (SD)	0.28 (0.38)	4.76 (9.92)	6.14 (12.35)	5.50 (6.61)	0.459	0.007	0.001	0.254	0.954
Total eosinophils (× 10^6/mL)	Mean (SD)	0.01 (0.02)	0.39 (1.17)	0.42 (0.66)	0.22 (0.22)	0.688	0.012	0.004	0.204	0.534
Sputum neutrophils, %	Mean (SD)	32.30 (24.35)	35.61 (25.92)	44.52 (27.99)	41.23 (25.64)	0.506	0.714	0.244	0.204	0.746
Total neutrophils (× 10^6/mL)	Mean (SD)	2.02 (2.41)	3.59 (5.40)	4.59 (7.07)	3.31 (4.84)	0.712	0.902	0.423	0.345	0.885
Total cell count (× 10^6/mL)	Mean (SD)	5.04 (3.48)	6.47 (6.23)	9.17 (8.54)	6.34 (4.73)	0.025	0.552	0.117	0.125	0.248
Asthma inflammatory phenotype (*N*, %)	Eosinophilic Non-eosinophilic	9 (100%)	14 (33%) 29 (67%)	10 (45%) 12 (55%)	6 (55%) 5 (45%)	0.037	0.092	0.030	0.226	0.451

Overall *P* values are from ANOVA or Kruskal–Wallis for numeric variables and Chi-squared test or Fisher’s exact test for categorical variables. *P* values for pairwise comparisons of numeric variables are from *T* tests or Wilcoxon-Mann–Whitney tests and are unadjusted for multiple testing. *ACQ-6* Asthma Control Questionnaire-6, *BMI* Body Mass Index, *FEV*_*1*_*preb2pp* Pre-Bronchodilator % Predicted FEV_1_, *FVCpreb2pp* Pre-Bronchodilator % Predicted FVC

### Isolation of peripheral blood mononuclear cells (PBMCs), NLRP3 inflammasome priming and activation and assessment of IL-1β release

PBMCs were isolated from whole blood and seeded at 2 × 10^5^ cells/well as outlined in the Supplementary Materials. They were then centrifuged prior to incubation in media and/or pre-treatment with lipopolysaccharide for 2 or 4 h (Additional file 1: Fig. S1A, B). LPS pre-treatment primes immune cells for NLRP3 inflammasome-induced IL-1β by inducing the intracellular production of NLRP3, pro-caspase-1 and pro-IL-1β [29–31]. PBMCs were then exposed to the NLRP3 inflammasome-activating compound, nigericin in the absence or presence of MCC950, for + 1 h (Additional file 1: Fig. S1A, B). Sham treatments for nigericin and MCC950 received PBS. Stimulated PBMCs were then centrifuged and culture supernatants collected for quantification of IL-1β by ELISA as outlined in the Supplementary Materials.

### Statistics

Group comparisons were between patients with asthma (classified as non-severe [stable], severe [stable], severe [exacerbating]) and healthy controls. The primary outcome IL-1β was measured 1 h following nigericin or sham treatment, which occurred 2 or 4 h after LPS or sham treatment. Experimental variables included 4 pre-treatments (Media, LPS, Nigericin, LPS + Nigericin) and 2 drug treatments with MCC950 (negative, positive). This was a ‘split plot’ design where a sample from each patient was analyzed under each combination of experimental variables (4 × 2 repeated measures per patient). The continuous outcome of IL-1β were analyzed with a Linear Mixed Model that included covariates for sex and obesity and a four-way interaction term for asthma group by the experimental design variables (pre-treatment*drug*asthma*timepoint) as well as all the lower order terms. The model was adjusted for within-person correlation of outcomes due to repeated measures and the optimal correlation structure (compound symmetry) was selected by comparing Akaike information criterion (AIC) values for different models. Linear mixed models were used to allow for the complex nature of the data and analysis, which included repeated measures on individual samples, different group sizes, some missing data values, and the need to adjust for covariates. Mixed models use all available data and produce robust estimates under a missing at random assumption.

Model effects and comparisons of interest were estimated via the restricted maximum likelihood method and presented as mean differences with 95% Confidence Intervals and associated p-values. Model validity was assessed via plots including studentized residuals *versus* fitted plots. Heteroscedasticity (non-constant variance) was present due to the extreme range of outcome values, so the robust variance estimator was used. Model *p*-values for selected comparisons were compared against those from non-parametric tests (Wilcoxon-Mann–Whitney) for model validation, producing broadly similar results.

## Results

### Nigericin-induced NLRP3 inflammasome-mediated IL-1β release is commonly increased in PBMCs from patients with severe and non-severe asthma

**Fig. 1:**
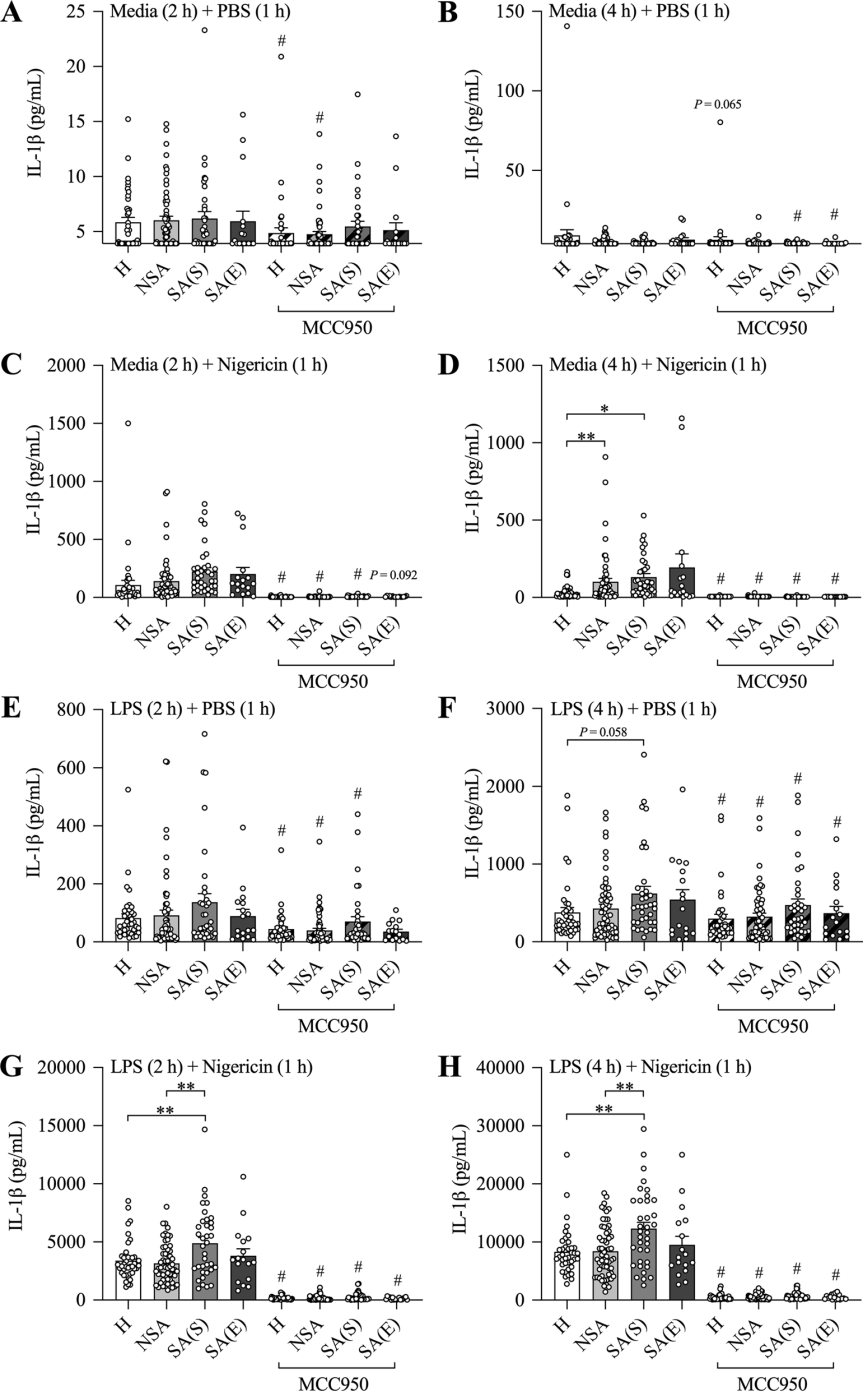
Peripheral blood mononuclear cells (PBMCs) from healthy subjects and patients with asthma differ in LPS- and nigericin-induced NLRP3 inflammasome–mediated IL-1β release. PBMCs from patients with non-severe asthma (NSA), severe asthma (stable SA(S); exacerbating SA(E)), and healthy (H) subjects were pre-treated with media (**A**–**D**) or LPS (**E**–**H**) for 2 (**A**, **C**, **E**, **G**) or 4 (**B**, **D**, **F**, **H**) hours before being treated with PBS (**A**, **B**, **E**, **F**) or Nigericin (**C**, **D**, **G**, **H**) for 1 h, and the effects of LPS and/or nigericin stimulation on IL-1β release was assessed in culture supernatants. Some cells were treated with MCC950 at the same time as PBS or nigericin stimulation to assess the effects of NLRP3 inflammasome inhibition on IL-1β release. Data are presented as means ± SEM (*N* = 17–59). ^*^P < 0.05, ^**^P < 0.01. ^#^*P* < 0.05 compared to control not treated with MCC950

The NLRP3 inflammasome is a multimeric protein signaling complex of the innate immune response that, upon activation, recruits, and proteolytically cleaves inactive pro-caspase-1 in active caspase-1 [27, 30–37]. Active caspase-1, in turn, cleaves inactive pro-IL-1β, to produce and release biologically active IL-1β [27, 30–37]. We first assessed the response of PBMCs from healthy subjects and patients with severe and non-severe asthma (Table 1) to NLRP3 inflammasome-induced IL-1β release. To do this we challenged PBMCs with the canonical NLRP3 activator, nigericin, which activates the NLRP3 inflammasome by inducing cellular potassium efflux [38]. Without nigericin treatment, there was no difference between IL-1β release from PBMCs from asthma patient groups compared to healthy subjects (Fig. 1A, B). However, following treatment with nigericin, PBMCs from patients with severe or non-severe asthma had increased IL-1β release compared to PBMCs from healthy subjects (Fig. 1C, D). Interestingly, there was no statistical difference between the amount of IL-1β released from PBMCs from severe and non-severe patients, and no difference between PBMCs from stable, compared to exacerbating, asthma patients. These findings suggest that increased sensitivity to nigerin-induced NLRP3 inflammasome activation-mediated IL-1β release is a common feature of systemic immune cells in patients with asthma.

### Nigericin-induced NLRP3 inflammasome-activation-mediated IL-1β release in PBMCs from patients with asthma is not affected by sex or obesity status and correlates with neutrophilic inflammation

**Fig. 2:**
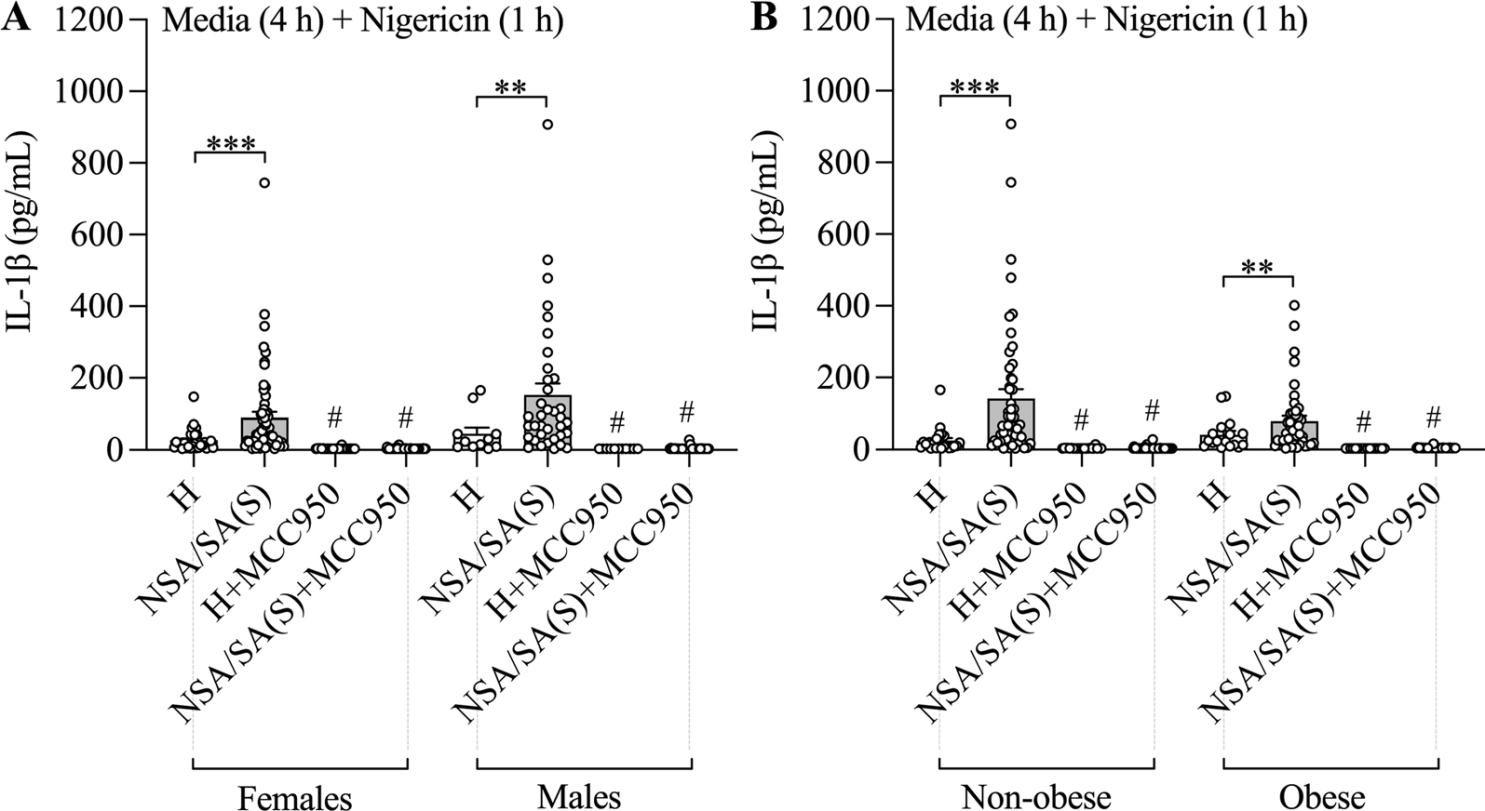
Nigericin-induced NLRP3 inflammasome-mediated IL-1β release in peripheral blood mononuclear cells (PBMCs) from patients with asthma is not affected by sex or obesity. PBMCs from patients non-severe (NSA) and severe stable (SA(S)) asthma and healthy (H) subjects were pre-cultured with media for 4 h before being stimulated with nigericin for 1 h, and the effects on IL-1β release assessed in culture supernatants. Some cells were treated with MCC950 at the same time as nigericin stimulation. The effects of nigericin stimulation and NLRP3 inflammasome inhibition on IL-1β release were assessed following stratification of subjects by sex **A** and obesity ) status. Data are presented as means ± SEM (*N* = 11–59). ^**^P < 0.01, ***P < 0.001. ^#^*P* < 0.05 compared to control not treated with MCC950

We next performed analyses to determine whether increased nigericin-induced NLRP3 inflammasome-mediated IL-1β release in asthma patients is different in males compared to females, or in obese compared to non-obese subjects. Nigericin-induced IL-1β release from PBMCs from all patients with asthma (severe and non-severe combined, as there was no difference in nigericin-induced IL-1β between these) was significantly increased compared to healthy subjects (Additional file 1: Tables S1 and S2, Fig. 2A, B). Stratification by sex and obesity status showed no effects on nigericin-induced responses. These findings show that increased nigericin-induced NLRP3 inflammasome-mediated IL-1β release occurs in both males and females as well as obese and non-obese subjects with asthma.

We next assessed correlations between on IL-1β release by nigericin-challenged PBMCs from all asthmatic subjects and BMI, asthma control (ACQ-6), sputum eosinophils and neutrophils (total, %) and pre-bronchodilator lung function (FEV_1_, FVC, FEV_1_/FVC) to determine whether increased NLRP3 inflammasome activation in PBMCs is associated with specific features of disease. Interestingly, nigericin-induced IL-1β release from PBMCs from asthma subjects positively correlated with neutrophil, but not eosinophil number or percentage in patient sputum, and negatively correlated with FEV_1_ and FVC, but not FEV_1_/FVC (Table 2). These findings suggest that increased pre-priming to NLRP3 inflammasome-mediated IL-1β release by systemic immune cells is associated with increased neutrophilic airway inflammation and lower lung function in asthma in our cohort comprised of patients with neutrophilic and eosinophilic disease phenotypes.

**Table 2:** Nigericin stimulation of peripheral blood mononuclear cells from patients with severe and non-severe asthma, Spearman correlation coefficients between IL-1β release and key clinical characteristics

	Media (2 h) + Nigericin (1 h)		Media (4 h) + Nigericin (1 h)	
BMI	*r* = − 0.14	*P* = 0.190	*r* = − 0.11	*P* = 0.299
ACQ-6, mean	*r* = 0.12	*P* = 0.258	*r* = 0.01	*P* = 0.892
Sputum eosinophils, %	*r* = − 0.11	*P* = 0.398	*r* = 0.05	*P* = 0.681
Sputum eosinophils total	*r* = 0.12	*P* = 0.352	*r* = 0.15	*P* = 0.253
Sputum neutrophils, %	*r* = 0.27	*P* = 0.030	*r* = 0.39	*P* = 0.001
Sputum neutrophils, total	*r* = 0.35	*P* = 0.006	*r* = 0.39	*P* = 0.002
FEV_1_preb2pp	*r* = − 0.24	*P* = 0.022	*r* = − 0.18	*P* = 0.088
FVCpreb2pp	*r* = − 0.21	*P* = 0.041	*r* = − 0.17	*P* = 0.095
FEV_1_/FVC, %	*r* = − 0.11	*P* = 0.303	*r* = − 0.16	*P* = 0.130

*ACQ-6* Asthma Control Questionnaire-6, *BMI* Body Mass Index, *FEV*_*1*_*preb2pp* Pre-Bronchodilator % Predicted FEV_1_, *FVCpreb2pp* Pre-Bronchodilator % Predicted FVC; *r* Spearman rho

### PBMCs from severe asthmatics have further increases in IL-1β production following LPS-induced priming and nigericin-induced NLRP3 inflammasome activation

NLRP3 inflammasome-mediated IL-1β responses involve two distinct signals. Signal one is the priming signal, which initiates the expression and translation of inflammasome components, such as NLRP3 and pro-caspase-1, along with pro-IL-1β and their assembly into a complex [29–31]. Signal two is the activating signal which initiates NLRP3 inflammasome-mediated cleavage and release of active IL-1β [29–31]. In order to further characterize NLRP3 inflammasome responses in immune cells from patients with asthma, we next assessed the release of IL-1β from PBMCs from healthy subjects, and patients with severe and non-severe asthma, following 2 and 4 h of pre-treatment with LPS (signal one)[29–31], prior to nigericin (signal two)-induced activation. LPS alone increased IL-1β release from PBMCs from all subject groups, however, there were no significant differences between any groups (Fig. 1E, F). However, following treatment with LPS + nigericin, PBMCs from patients with severe asthma released increased IL-1β levels compared to PBMCs from patients with non-severe asthma and healthy subjects (Fig. 1G, H). Interestingly, there were no differences between the amount of IL-1β released from PBMCs from non-severe patients and healthy subjects or stable compared to exacerbating severe asthma patients. Together, these findings demonstrate that, whilst systemic immune cells from asthma patients are more sensitive to nigericin-induced inflammasome activation (signal two), an increased response to both LPS-induced priming (signal one) and nigericin-induced activation (signal two) of the NLRP3 inflammasome is a unique feature of systemic immune cells in patients with severe asthma. This increased response to LPS-induced priming and nigericin-induced activation is not affected during exacerbations of severe asthma.

### Increased LPS-induced NLRP3 inflammasome priming and nigercin-induced activation in PBMCs from patients with severe asthma compared to other subjects is not affected by sex or obesity status and correlates with both neutrophilic and eosinophilic inflammation

**Fig. 3:**
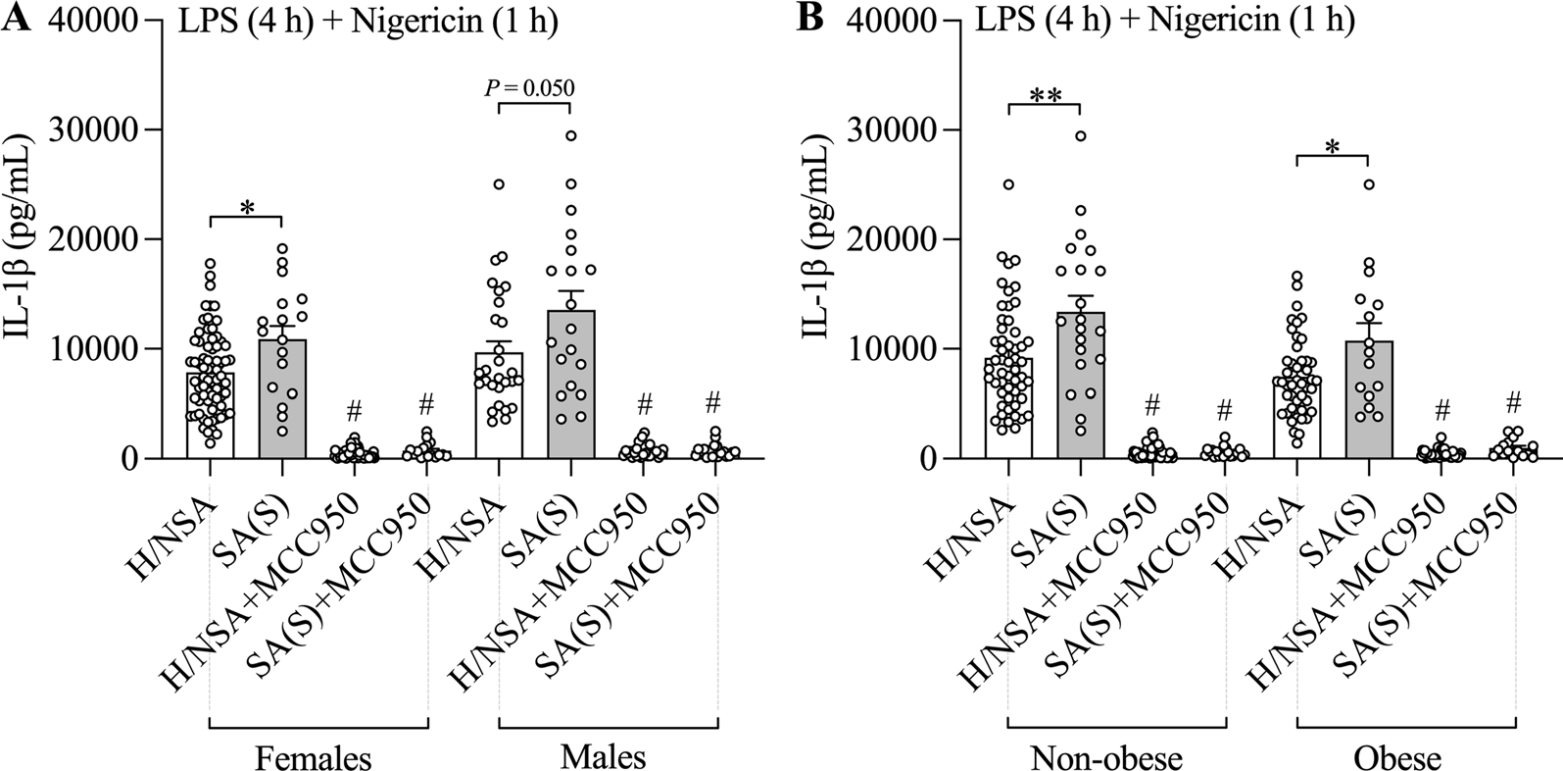
Increased LPS-induced NLRP3 inflammasome priming and nigercin-induced activation in peripheral blood mononuclear cells (PBMCs) from patients with severe asthma compared to other subjects is not affected by sex or obesity. PBMCs from patients with non-severe (NSA) and severe stable (SA(S)) asthma, and healthy (H) subjects were pre-stimulated with LPS for 4 h before stimulation with nigericin for 1 h, and the effects of LPS + Nigericin on IL-1β release was assessed in culture supernatants. Some cells were treated with MCC950 at the same time as nigericin stimulation. The effects of LPS + Nigericin stimulation and NLRP3 inflammasome inhibition on IL-1β release were assessed in patients with SA(S) compared to other subjects (H and NSA together) following stratification of subjects by sex **A** and obesity **B** status. Data are presented as means ± SEM (*N* = 15–70). ^*^P < 0.05, ^**^P < 0.01. ^#^*P* < 0.05 compared to control not treated with MCC950

We next performed analyses to determine whether increased IL-1β release induced by LPS + nigericin treatment severe asthma is different in males compared to females, or in obese compared to non-obese subjects. LPS + nigericin-induced IL-1β release from PBMCs from patients with severe asthma was significantly increased compared to other participant groups (non-severe asthma and healthy subjects combined, as there was no difference in LPS + nigericin-induced IL-1β between these groups) and stratification by sex (Additional file 1: Tables S3 and S4, Fig. 3A) or obesity status (Additional file 1: **Tables S3 and S4, **Fig. 3B**) had no effects**. These findings suggest that increased LPS-induced NLRP3 inflammasome priming and nigericin-induced activation occurs in both males and females as well as in obese and non-obese subjects with severe asthma.

We next assessed correlations between on IL-1β release by LPS + nigericin-treated PBMCs from patients with severe asthma and BMI, control, eosinophilic and neutrophilic inflammation in sputm and lung function, to determine whether increased LPS-induced NLRP3 inflammasome priming and nigercin-induced activation in PBMCs is associated with specific features of disease. LPS + nigericin-induced IL-1β release from PBMCs from patients with severe asthma positively correlated with both total eosinophilic and neutrophilic inflammatory cell numbers in sputum (Table 3). These findings suggest that increased LPS-induced NLRP3 inflammasome priming and nigericin-induced activation in systemic immune cells is associated with both eosinophilic and neutrophilic inflammatory responses in the airways of patients with severe asthma.

**Table 3:** LPS + Nigericin stimulation of peripheral blood mononuclear cells from patients with severe asthma, Spearman correlation coefficients between IL-1β release and key clinical characteristics

	LPS (2 h) + Nigericin (1 h)		LPS (4 h) + Nigericin (1 h)	
BMI	*r* = − 0.13	*P* = 0.436	*r* = − 0.27	*P* = 0.113
ACQ-6, mean	*r* = 0.12	*P* = 0.489	*r* = − 0.00	*P* = 0.980
Sputum eosinophils, %	*r* = − 0.17	*P* = 0.456	*r* = 0.26	*P* = 0.247
Sputum eosinophils, total	*r* = 0.46	*P* = 0.043	*r* = 0.55	*P* = 0.012
Sputum neutrophils, %	*r* = 0.40	*P* = 0.067	*r* = 0.18	*P* = 0.415
Sputum neutrophils, total	*r* = 0.48	*P* = 0.032	*r* = 0.33	*P* = 0.158
FEV_1_preb2pp	*r* = 0.17	*P* = 0.339	*r* = 0.12	*P* = 0.478
FVCpreb2pp	*r* = 0.13	*P* = 0.473	*r* = 0.14	*P* = 0.435
FEV_1_/FVC, %	*r* = 1.00	*P* = 0.568	*r* = 0.06	*P* = 0.741

*ACQ-6* Asthma Control Questionnaire-6, *BMI* Body Mass Index, *FEV*_*1*_*preb2pp* Pre-Bronchodilator % Predicted FEV_1_, *FVCpreb2pp* Pre-Bronchodilator % Predicted FVC, *LPS* Lipopolysaccharide, *r* Spearman rho

### MCC950 reduces LPS- and NLRP3 inflammasome-induced IL-1β release from PBMCs with the greatest effects in those with severe asthma

Finally, we determined the effectiveness of MCC950 treatment in reducing NLRP3 inflammasome-mediated IL-1β release from PBMCs from patients with asthma. MCC950, potently suppressed IL-1β release from PBMCs from patients with severe (stable and exacerbating) and non-severe asthma and healthy subjects that are stimulated with nigericin alone, LPS alone, or LPS + nigericin (Fig. 1A–H). We show that the greatest effects of MCC950 suppression on NLRP3 inflammasome-mediated IL-1β release occurs in PBMCs from severe asthmatics (Table 4). Furthermore, we show that MCC950 effectively suppressed IL-1β release following stimulation with nigericin alone and LPS + nigericin in males and females as well as obese and non-obese subjects (Additional file 1: Tables S5 and S6). Together these findings indicate that MCC950 potently suppresses NP3 inflammasome-mediated IL-1β responses in systemic immune cells from all patients with asthma irrespective of disease severity and sex or obesity status.

**Table 4:** Effect size of MCC950 on IL-1β secretion from peripheral blood mononuclear cells

	Non-severe vs Healthy	Severe vs Healthy	Severe vs Non-severe	Severe vs Severe (exacerbating)
Media (2 h)				
Mean diff. (95% CI)	− 0.3 (− 1.1 to 0.5)	0.3 (− 1.0 to 1.6)	0.5 (− 0.7 to 1.8)	0.1 (− 1.5 to 1.7)
*P* value	0.5016	0.6882	0.3812	0.9070
LPS (2 h)				
Mean diff. (95% CI)	− 13.5 (− 40.2 to 13.3)	− 28.2 (− 59.2 to 2.8)	− 14.7 (− 51.8 to 22.3)	− 68.8 (− 178.6 to 41.0)
*P* value	0.3244	0.0755	0.4355	0.2202
Nigericin (1 h)				
Mean diff. (95% CI)	− 32.2 (− 120.9 to 56.5)	− 113.9 (− 213.6 to − 14.2)	− 81.7 (− 162.7 to − 0.8)	− 76.0 (− 251.7 to 99.7)
*P* value	0.4773	0.0256	0.0484	0.3970
LPS (2 h) + Nigericin (1 h)				
Mean diff. (95% CI)	240.2 (− 412.3 to 892.7)	− 1380.9 (− 2399.2 to − 362.5)	− 1621.0 (− 2589.7 to − 652.4)	− 937.6 (− 2363.3 to 488.1)
*P* value	0.4710	0.0082	0.0011	0.1981
Media (4 h)				
Mean diff. (95% CI)	− 2.4 (− 11.6 to 6.8)	2.2 (− 0.9 to 5.4)	4.6 (− 4.0 to 13.3)	1.5 (− 0.4 to 3.5)
*P* value	0.6096	0.1600	0.2948	0.1293
LPS (4 h)				
Mean diff. (95% CI)	− 30.3 (− 74.4 to 13.8)	− 66.8 (− 128.9 to − 4.7)	− 36.5 (− 99.4 to 26.4)	29.5 (− 87.9 to 146.8)
*P* value	0.1783	0.0357	0.2560	0.6229
Nigericin (1 h)				
Mean diff. (95% CI)	− 73.6 (− 118.2 to − 29.1)	− 99.7 (− 143.4 to − 56.1)	− 26.1 (− 86.0 to 33.8)	62.0 (− 109.8 to 233.8)
*P* value	0.0013	< 0.0001	0.3933	0.4799
LPS (4 h) + Nigericin (1 h)				
Mean diff. (95% CI)	− 79.3 (− 1646.5 to 1487.9)	− 3735.3 (− 6046.7 to − 1423.8)	− 3655.9 (− 5915.2 to − 1396.7)	103.4 (38.1 to 168.8)
*P* value	0.9210	0.0016	0.0016	0.0020

*LPS* Lipopolysaccharide

Descriptive statistics are shown as mean difference (95% confidence interval)

## Discussion

**Fig. 4:**
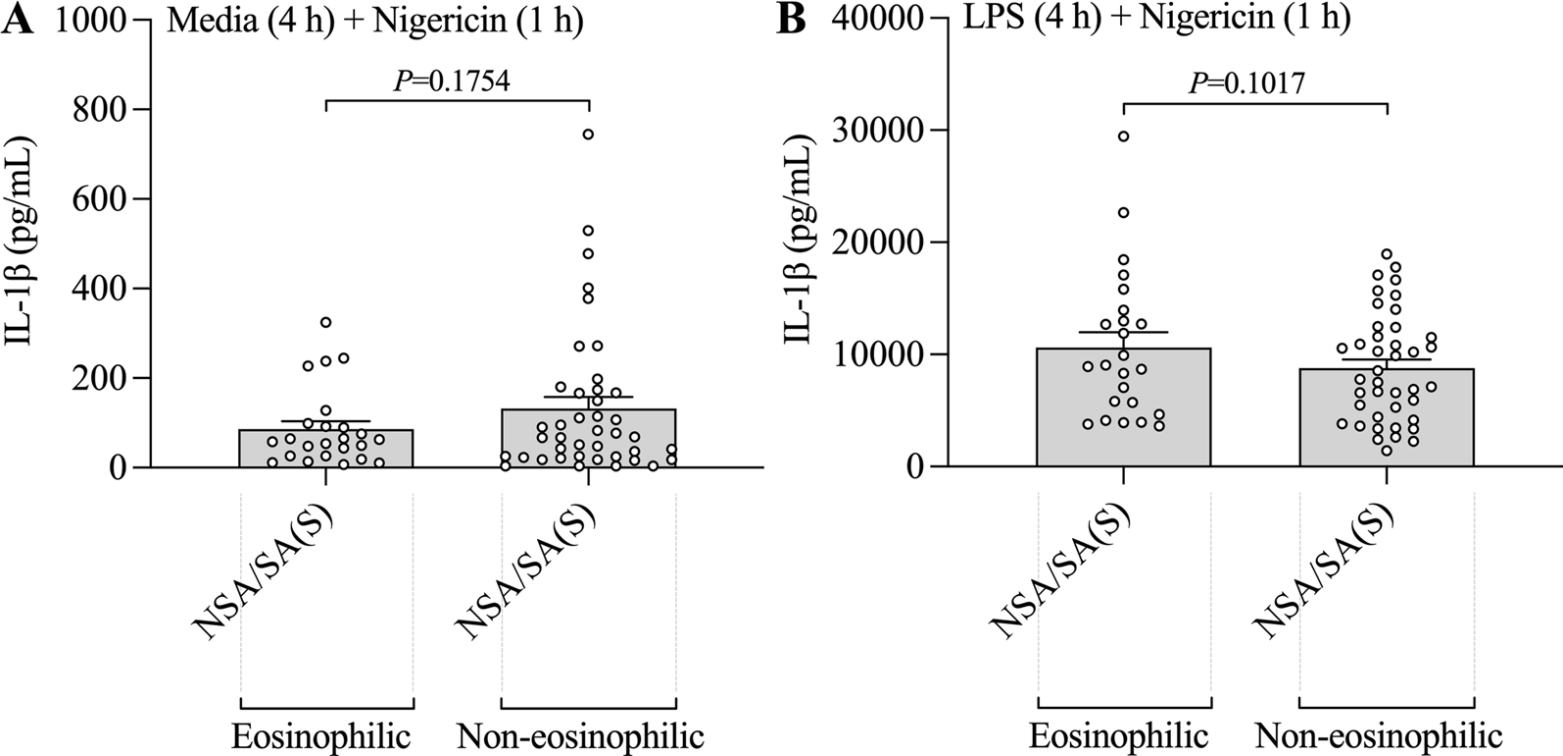
Increased LPS-induced and/or nigercin-induced NLRP3 inflammasome-mediatedIL-1β release in peripheral blood mononuclear cells (PBMCs) from patients with asthma is not affected by sputum inflammatory phenotype. PBMCs from patients non-severe (NSA) and severe stable (SA(S)) asthma were **A** pre-cultured with media for 4 h before being stimulated with nigericin for 1 h, or **B** pre-stimulated with LPS for 4 h before stimulation with nigericin for 1 h, and the effects on IL-1β release were assessed in culture supernatants. The effects of nigericin or LPS + Nigericin stimulation on IL-1β release were assessed following stratification of subjects by sputum inflammatory phenotype. Data are presented as means ± SEM (*N* = 24–41)

We, and others, previously showed that NLRP3 inflammasome and IL-1β responses are increased in the airways of patients with severe and neutrophilic asthma [19–26]. Here, we show that systemic immune cells from all patients with asthma, both severe and non-severe asthmatics that contain both eosinophilic and non-eosinophilic populations, have an increased ability for nigericin-induced NLRP3 inflammasome activation compared to those from non-asthma subjects (Fig. 1D). NLRP3 inflammasome activation has been shown to play a critical role in breaking tolerance to antigens and the induction of experimental asthma [39, 40]. Our findings provide clinical evidence that an increased ability for inflammasome activation in systemic immune cells may play a crucial role in the pathogenesis of inflammatory processes that underpin both severe and non-severe asthma. After puberty, females are more likely to have asthma than males (60% *versus* 40% of non-severe asthma)[41] and up to 82% of patients with severe asthma are female [42]. Furthermore, we, and others, have identified severe asthma in obese women as a distinct clinical phenotype that is associated with increased NLRP3 inflammasome responses in the airways [6, 13–16]. Here, we show that inflammasome responses are not increased in systemic immune cells from female *versus* male, or obese *versus* non-obese, people with asthma. Interestingly, we did see a small increase in inflammasome responses in systemic immune cells from obese non-asthmatic subjects compared to non-obese controls, but this did not reach statistical significance. We also show that there are no differences between nigericin- or LPS + nigericin-induced IL-1β release in PBMCs from asthmatic subjects (severe and non-severe subjects combined) with eosinophilic *versus* non-eosinophilic asthma (Additional file 1: Table S7 and Fig. 4). This suggests that the increased ability for activation of inflammasome responses that we have identified may be universal features of systemic immune cells in patients with severe and non-severe asthma (*i.e.* all asthma) and are not dependent upon sex or obesity status, or sputum granulocyte composition. Together, these findings highlight that an increased ability for activation of inflammasome responses in systemic immune cells may play a fundamental underlying role in asthma.

We also show that systemic immune cells from patients with severe asthma release more IL-1β following a combination of pathogen (LPS)-induced priming and NLRP3 inflammasome activation compared to cells from patients with non-severe asthma (Fig. 1G, H). These findings indicate a potential fundamental difference between severe and non-severe asthma. They demonstrate that systemic immune cells from patients with severe asthma have an increased ability to respond to pathogen component-induced priming step required to produce inflammasome components and pro-IL-1β, in addition to increased ability to respond to inflammasome activation required to cleave and release active IL-1β. Microbial infections are an important stimulus for NLRP3 inflammasome priming and activation in the lung [27, 38–45]. We previously showed that respiratory infections have important roles in initiating immune responses, including NLRP3 inflammasome-mediated IL-1β responses, in the asthmatic lung that promote severe, steroid-insensitive asthma [6, 27, 44, 45]. Our current findings demonstrate that systemic immune cells in severe asthmatics may also be more responsive to infection-induced priming and subsequent NLRP3 inflammasome-mediated, steroid-insensitive inflammatory responses. Our clinical findings highlight a potential mechanism to explain the link between infection and the induction of heightened inflammatory processes that underpin severe asthma.

Furthermore, we show that increased LPS + nigericin-induced NLRP3 inflammasome-mediated IL-1β release from PBMCs from patients with severe asthma correlate with increased total eosinophil and neutrophil numbers in the airways (Table 3). This suggests that the responses we have identified are associated with both eosinophilic, and neutrophilic, inflammatory responses in severe asthma. We previously showed that respiratory infections influence the nature of inflammatory responses in experimental severe asthma with bacterial infections promoting neutrophilic, and viral infections promoting eosinophilic, inflammation [45]. Based upon these findings, we propose that systemic immune cells in patients with severe asthma may have increased sensitivity to inflammasome priming and activation to different triggers and that the nature of the inflammasome-activating stimuli in the airways upon recruitment, plays the crucial role in determining the nature of the inflammatory phenotype in severe asthma. Interestingly, we show that immune cells have similar responses in stable and exacerbating severe asthmatics. This suggests that the heightened responsiveness to infection-induced priming of inflammasome-mediated responses in severe asthmatics when stable may underpin the increased risk of viral and/or bacterial infection-induced exacerbations in patients with severe asthma [43, 46, 47].

We previously showed that increased IL-1β responses drive steroid-insensitive, inflammation and AHR, and that inhibiting NLRP3 activation with MCC950 reduced IL-1β production and ablated these features in murine models of severe asthma [27]. We now show that increased NLRP3 inflammasome-mediated IL-1β release from immune cells from humans with severe asthma can be pharmacologically inhibited with MCC950, demonstrating therapeutic potential for inflammasome inhibition in clinical settings. We show that increased ability for inflammasome activation is common to severe and non-severe asthma, in male and female, and obese and non-obese, individuals and is associated with neutrophilic and eosinophilic inflammation in severe asthma. Thus, therapeutic strategies that target inflammasome priming and/or activation may represent a new, broadly applicable approach to asthma management, particularly in severe, T2-low subtypes of disease.

It should be noted that there is a significant difference between the ages of the SA, NSA and healthy control groups. These data agree with previous findings that show that older asthmatics are more likely to have increased disease severity. Whilst our data clearly show increases in inflammasome-mediated IL-1β release from PBMCs from asthmatics, a link between increased IL-1β release from PBMCs and age cannot be ruled out and requires investigation in future studies. A limitation of the current study is that we performed all assessments in systemic immune cells. Whilst we were not able to conduct our NLRP3 inflammasome stimulation studies on cells isolated from the airways of participants, it is also likely that inflammasome responses would have already been activated to varying degrees in these cells and this would convolute the interpretation of the outcomes and compromise the relevance of the findings. We have previously shown that airways NLRP3 inflammasome responses are associated with increased neutrophilic and eosinophilic inflammation in the airways in both experimental and clinical asthma [16, 27], and there are subjects with asthma and IL-5-, IL-17A-/F- and IL-25-high sputum cytokine profiles that have increased sputum eosinophil and neutrophil numbers [48, 49]. These findings highlight the co-existence of T2 and non-T2 cytokine responses in the airways with both eosinophilic and non-eosinophilic airways inflammation. Our data in the current study, that have been generated from PBMCs, extend upon these findings to support a fundamental underlying role for an increased ability of NLRP3 inflammasome responses in systemic immune cells in being associated with both eosinophilic and neutrophilic inflammatory cell numbers in the airways. Indeed, our data highlight that, as opposed to inflammatory cells and responses in the airways, systemic cells may be more homogenous in their behavior with a critical role for inflammasome responses underpinning both eosinophilic and non-eosinophilic asthma.

Another limitation of the study was that the cellular source and intracellular mechanisms of inflammasome signaling and IL-1β release from different cells were not fully characterized. We also did not perform a more detailed characterization of immune responses beyond eosinophilic and neutrophilic inflammation to provide a more thorough delineation between T2 and non-T2 asthma. Unfortunately, such analyses were not feasible in the current study, which was designed to assess the differences of NLRP3 inflammasome priming and activation, and effectiveness of inflammasome inhibition, in systemic immune cells in asthma. Nevertheless, our study, which investigated responses in a large number of subjects, clearly shows increased inflammasome responses, and highlights the potential for therapeutic targeting, in systemic immune cells in all subtypes of asthma. Our findings are novel and will spur future studies that identify the cellular source(s) of altered inflammasome responses and fully characterize which components of the inflammasome signaling network are altered in these cells as well as further interrogate these responses in T2 and non-T2 asthma. Importantly, our study shows strong relationships between IL-1β release from PBMCs and asthma status. These studies highlight the utility of investigating cytokine responses from PBMCs and other systemic immune cells for better understanding the immunobiology of asthma. Of note, through minor alterations to the assay system employed in the current study IL-17, which is an example of a cytokine known to be related to neutrophilic endotypes of asthma, release from PBMCs of subjects with eosinophilic and non-eosinophilic asthma could be explored.

We have previously shown that NLRP3 inflammasome-mediated IL-1β responses in the airways play a key role in the pathogenesis of severe neutrophilic asthma. The current study extends upon these findings by demonstrating, for the first time, that systemic immune cells in clinical asthma have an increased ability for inflammasome-mediated IL-1β release, regardless of asthma subtype. Importantly, we highlight that NLRP3 inflammasome-mediated IL-1β responses can be therapeutically suppressed in systemic immune cells from patients across all subtypes of both severe and non-severe asthma.

### Supplementary Information

**Additional file 1.** Additional methods and materials, tables and figures.

## Data Availability

Not applicable.

## Abbreviations

LPS: Lipopolysaccharide
NLRP3: NLR family, pyrin domain–containing (NLRP) 3 inflammasome
PBMC: Peripheral blood mononuclear cell
T2: Type 2
