# Supplementary material for: Characterization and inhibition of inflammasome responses in severe and non-severe asthma
Source: Respir Res. 2023 Dec 4;24:303. doi: 10.1186/s12931-023-02603-2 (PMC10694870; doi:10.1186/s12931-023-02603-2)
Supplement: Supplementary file 1 — Additional file 1. Additional methods and materials, tables and figures. [file 12931_2023_2603_MOESM1_ESM.docx]

**Online Repository**

**Characterization and inhibition of inflammasome responses in severe and non-severe asthma**

Jay C. Horvat, PhD,^1†*^ Richard Y. Kim, PhD,^1,2†^ Natasha Weaver, PhD,^1†^ Christopher Augood, BBiomedSci(Hons),^1,3^ Alexandra C. Brown, PhD,^1^ Chantal Donovan, PhD,^1,2^ Pierrick Dupre, MSc,^1,4^ Lakshitha Gunawardhana, PhD,^1^ Jemma R. Mayall, PhD,^1^ Nicole G. Hansbro, PhD,^1,3^ Avril A. B. Robertson, PhD,^5^ Luke A. J. O’Neill, PhD,^6^ Matthew A. Cooper, PhD,^7^ Elizabeth G. Holliday, PhD,^1^ Philip M. Hansbro, PhD,^1,3^ and Peter G. Gibson, MBBS, FRACP,^1^

**Affiliations:**

^1^University of Newcastle and Hunter Medical Research Institute, Newcastle, New South Wales, Australia.

^2^School of Life Sciences, Faculty of Science, University of Technology Sydney, Sydney, Australia.

^3^Centre for Inflammation, Centenary Institute and University of Technology Sydney, School of Life Sciences, Faculty of Science, Sydney, Australia.

^4^Montpellier Cancer Research Institute (IRCM), University of Montpellier, Montpellier, France.

^5^School of Chemistry and Molecular Biosciences, The University of Queensland, Brisbane, Australia.

^6^School of Biochemistry and Immunology, Trinity Biomedical Sciences Institute, Trinity College Dublin, Dublin, Ireland.

^7^Sitala Bio Ltd., Unit D6 Grain House, Mill Court, Great Shelford, CB22 5LD, UK.

^*^Correspondence to: Jay Horvat, Hunter Medical Research Institute, Lot 1 Kookaburra Circuit, New Lambton Heights, Newcastle, New South Wales, 2305, Australia. Email: [Jay.Horvat@newcastle.edu.au](mailto:Jay.Horvat@newcastle.edu.au)

^†^Authors contributed equally to this manuscript.

**MATERIALS AND METHODS**

**Study population**

The study sample comprised 151 adult participants (≥18 years) divided into 4 diagnostic subgroups: 39 healthy controls, 59 patients with non-severe (mild-moderate, stable) asthma, 36 patients with severe stable and 17 with severe exacerbating asthma (**Table 1**). Participants were recruited from the John Hunter Hospital Ambulatory Care Clinic, Newcastle, Australia. Asthma was diagnosed according to American Thoracic Society guidelines based on current (past 12 months) episodic respiratory symptoms, doctor’s diagnosis (ever), and documented evidence of variable airflow obstruction at screening or in the 10 years prior (FEV_1_ bronchodilator response >200mL or >12%, or AHR in response to any standard challenge, or peak flow variability >12% over >1 week, or variability >12% between 2 FEV_1_ values measured within 2 months of each other). Current and ex-smokers were excluded. Patients with post-bronchodilator FEV_1_<40% predicted, respiratory disease other than asthma, current pregnancy, current lung cancer or other blood, lymphatic or solid malignancy were also excluded. Exclusion criteria for stable asthma included recent (past 4 weeks) respiratory infection, asthma exacerbation, ED visit for asthma, unstable asthma (Asthma Control Questionnaire [ACQ]6 increase ≥0.5) or change in maintenance therapy. ***Non severe-asthma (NSA)*** was defined as GINA treatment step 1-4 (no ICS or low-medium dose ICS). ***Severe asthma (SA)*** was defined as uncontrolled [(Juniper ACQ>1.5), and/or ≥2 exacerbations requiring prednisone in the past year (>10mg, >3 days), and/or ≥1 serious exacerbation (hospitalization, ICU stay or mechanical ventilation in past year), and/or persistent airflow limitation (pre-bronchodilator FEV_1_% < 80)] asthma, despite adherence to high dose ICS (>1,000μg beclomethasone equivalent daily) in combination with a second controller. ***Exacerbating SA*** (assessed within the 4 week exacerbation window) is SA requiring treatment with prednisone (>10mg for >3 days) or antibiotics, or ED visit, or increased ACQ6≥0.5, or increase in asthma medications. ***Eosinophilic asthma*** is defined as asthma with ≥3% sputum eosinophils and ***non-eosinophilic asthma*** defined as asthma with <3% sputum eosinophils. Clinical assessments and data collection included demographics, medical, asthma and exacerbation history (past 12 months), medication use, asthma symptom control (Juniper ACQ6), lung function and saline-induced sputum induction. 100% of the subjects with asthma (NSA or SA) were on inhaled corticosteroids. None of the subjects with stable asthma (NSA or SA) or healthy controls were on oral corticosteroids (OCS). 100% of the subjects with exacerbating SA were on OCS. Sputum samples were induced and processed as previously described.^28^

**Isolation of peripheral blood mononuclear cells (PBMCs) from whole blood**

Whole blood from each participant was collected (including at the time of exacerbation) into 9mL VACUETTE™ K3EDTA tubes (Greiner Bio-One), combined 1:1 with warm Dulbecco′s Phosphate Buffered Saline (PBS; #D8537 Sigma-Aldrich), gently overlayed onto 10mL of Ficoll® Paque Plus (#GE17-1440-02 Cytiva), and centrifuged (430 x*g*, 30 mins, 22°C). PBMCs were harvested from the interface between Ficoll® Paque Plus and plasma, supplemented with warm PBS (40mL total volume), centrifuged (170 x*g*, 10 mins, 22°C), and the supernatant discarded. PBMCs were then resuspended in 10mL of warm PBS, centrifuged (170 x*g*, 10 mins, 22°C), and the supernatant discarded. Purified PBMCs were resuspended in 1mL of complete RPMI (Sigma-Aldrich, Sydney, Australia) and viable cells enumerated by trypan blue dye exclusion.

***Ex vivo* PBMC LPS-induced NLRP3 inflammasome priming and nigericin-induced activation culture conditions**

PBMCs were seeded at 2x10^5^ cells/well in RPMI (Sigma-Aldrich, Sydney, Australia) supplemented with 1mM Na-Pyruvate (Sigma-Aldrich, Sydney, Australia), 2mM L-glutamine (Gibco, Tullamarine, Australia), 10mM HEPES (Gibco, Tullamarine, Australia), 5U/mL Penicillin (Gibco, Tullamarine, Australia), 5ug/ml Streptomycin (Gibco, Tullamarine, Australia) and 50µM 2-mercaptoethanol (Sigma-Aldrich, Sydney, Australia). They were then centrifuged (170 x*g*, 10 mins, 22°C) prior to incubation (37°C, 5% CO_2_) in media and/or pre-treatment with lipopolysaccharide (LPS; 1μg/mL, L4524, Sigma-Aldrich, Sydney, Australia) for 2 or 4 hours (**Figures E1A and E1B**). LPS pre-treatment primes immune cells for NLRP3 inflammasome-induced IL-1β by inducing the intracellular production of NLRP3, pro-caspase-1 and pro-IL-1β.^29-31^ PBMCs were then exposed to the NLRP3 inflammasome-activating compound, nigericin (5μM; N7143, Sigma-Aldrich, Sydney, Australia) in the absence or presence of MCC950, for +1 hour (**Figures E1A and E1B**). Sham treatments for nigericin and MCC950 received PBS. All stimulation conditions were assayed in triplicate. Stimulated PBMCs were then centrifuged (430 x*g*, 5 mins, 22°C) and culture supernatants collected for quantification of IL-1β.

**Enzyme-linked immunosorbent assay (ELISA) for IL-1β release from *ex vivo* PBMC cultures**

IL-1β protein levels in PBMC culture supernatants, which represent the active form of IL-1β that is released following stimulation, were measured by ELISA using Human IL-1 beta/IL-1F2 DuoSet ELISA kits (DY201, R&D Systems, MN, USA). Briefly, 96-well flat-bottomed high binding ELISA plates (Corning Inc., Corning, USA) were coated with mouse anti-Human IL-1β (R&D Systems, MN, USA; 25μL, 4μg/mL, room temperature [RT] overnight). Coated plates were washed (3x0.05% PBS-Tween 20 [PBS-T]), blocked (1% BSA Sigma-Aldrich, Sydney, Australia in 150μL PBS, RT, 60 mins) and washed again (3xPBS-T). Samples (50μL of neat or 1:10 or 1:100 dilutions of PBMC culture supernatant) and standards (recombinant human IL-1β, R&D Systems, MN, USA; 50μL in 1% BSA in PBS; 250pg/mL – 3.91pg/mL range) were then added in duplicate and plates incubated (RT, 120 mins). Plates were then washed (3xPBS-T), coated with biotinylated goat anti-Human IL-1β (R&D Systems, MN, USA; 50μL, 150ng/ml, RT, 120 mins), washed (3xPBS-T then 2xPBS), and streptavidin-HRP conjugate added (R&D Systems, MN, USA; 50μL, 1:40 dilution in 1% BSA in PBS, RT, 20 mins). Plates were again washed (3xPBS-T, 2xPBS). Colorimetric reactions were developed by incubation with tetramethylbenzidine (50μL, 0.1mg/mL in citrate buffer, RT, 20 mins in the dark, Sigma-Aldrich, Sydney, Australia) and reactions were terminated with sulfuric acid (25μL; 2N). A microplate spectrophotometer (SpectraMax M, Molecular Devices, CA, USA) was used to read optical absorbances at 450nm and concentrations of IL-1β in samples calculated by interpolation with the standard curve.

**Table E1.** Nigericin-induced IL-1β secretion from peripheral blood mononuclear cells stratified based on sex

|  | **Females**  **(Severe/Non-severe vs Healthy)** | | **Males**  **(Severe/Non-severe vs Healthy)** | |
| --- | --- | --- | --- | --- |
|  | **Mean diff (95% CI)** | ***P* Value** | **Mean diff (95% CI)** | ***P* Value** |
| Media (2 h) + Nigericin (1 h) | 45.9 (-68.6 to 160.3) | 0.4327 | 115.4 (39.7 to 191.1) | 0.0033 |
| Media (4 h) + Nigericin (1 h) | 62.4 (29.4 to 95.3) | 0.0003 | 107.2 (38.7 to 175.7) | 0.0026 |

Descriptive statistics are shown as mean difference (95% confidence interval).

**Table E2.** Nigericin-induced IL-1β secretion from peripheral blood mononuclear cells stratified based on obesity status

|  | **Non-obese**  **(Severe/Non-severe vs Healthy)** | | **Obese**  **(Severe/Non-severe vs Healthy)** | |
| --- | --- | --- | --- | --- |
|  | **Mean diff (95% CI)** | ***P* Value** | **Mean diff (95% CI)** | ***P* Value** |
| Media (2 h) + Nigericin (1 h) | 175.8 (86.0 to 265.6) | 0.0002 | -0.2 (-131.1 to 130.6) | 0.9970 |
| Media (4 h) + Nigericin (1 h) | 114.5 (59.4 to 169.5) | <0.0001 | 64.6 (23.5 to 105.7) | 0.0023 |

Descriptive statistics are shown as mean difference (95% confidence interval).

**Table E3.** LPS+Nigericin-induced IL-1β secretion from peripheral blood mononuclear cells stratified based on sex

|  | **Females**  **(Severe vs Non-severe/Healthy)** | | **Males**  **(Severe vs Non-severe/Healthy)** | |
| --- | --- | --- | --- | --- |
|  | **Mean diff (95% CI)** | ***P* Value** | **Mean diff (95% CI)** | ***P* Value** |
| LPS (2 h) + Nigericin (1 h) | 1610.1 (520.7 to 2699.5) | 0.0041 | 1429.7 (-296.5 to 3155.8) | 0.1069 |
| LPS (4 h) + Nigericin (1 h) | 3022.3 (565.7 to 5478.9) | 0.0166 | 3852.8 (28.3 to 7677.2) | 0.0504 |

Descriptive statistics are shown as mean difference (95% confidence interval).

**Table E4.** LPS+Nigericin-induced IL-1β secretion from peripheral blood mononuclear cells stratified based on obesity status

|  | **Non-obese**  **(Severe vs Non-severe/Healthy)** | | **Obese**  **(Severe vs Non-severe/Healthy)** | |
| --- | --- | --- | --- | --- |
|  | **Mean diff (95% CI)** | ***P* Value** | **Mean diff (95% CI)** | ***P* Value** |
| LPS (2 h) + Nigericin (1 h) | 2572.5 (1010.6 to 4134.4) | 0.0017 | 1296.2 (73.0 to 2519.5) | 0.0387 |
| LPS (4 h) + Nigericin (1 h) | 6624.6 (1948.8 to 11300.4) | 0.0065 | 2888.1 (564.1 to 5212.1) | 0.0155 |

Descriptive statistics are shown as mean difference (95% confidence interval).

**Table E5.** Effect (positive *vs* negative) of MCC950 on nigericin- and LPS+Nigericin-induced IL-1β secretion from peripheral blood mononuclear cells stratified based on sex

|  | **Females** | | | | **Males** | | | |
| --- | --- | --- | --- | --- | --- | --- | --- | --- |
|  | **Healthy/Non-severe** | | **Severe** | | **Healthy/Non-severe** | | **Severe** | |
|  | **Mean diff (95% CI)** | ***P* Value** | **Mean diff (95% CI)** | ***P* Value** | **Mean diff (95% CI)** | ***P* Value** | **Mean diff (95% CI)** | ***P* Value** |
| Media (2 h) + Nigericin (1 h) | -124.1  (-176.9 to  -71.3) | <0.0001 | -243.7  (-328.1 to  -159.3) | <0.0001 | -114.6  (-175.5 to  -53.8) | 0.0003 | -191.3  (-288.8 to  -93.8) | 0.0002 |
| Media (4 h) + Nigericin (1 h) | -56.3  (-81.5 to  -31.2) | <0.0001 | -112.6  (-158.7 to  -66.5) | <0.0001 | -110.2  (-178.1 to  -42.3) | 0.0018 | -141.4  (-208.7 to  -74.0) | <0.0001 |
| LPS (2 h) + Nigericin (1 h) | -2925.6  (-3253.5 to  -2597.8) | <0.0001 | -4406.0  (-5334.9 to -3477.1) | <0.0001 | -3473.9  (-4201.5 to  -2746.3) | <0.0001 | -4789.4  (-6230.5 to  -3348.4) | <0.0001 |
| LPS (4 h) + Nigericin (1 h) | -7408.8  (-8218.5 to  -6599.1) | <0.0001 | -10140.6  (-12222.4 to -8058.8) | <0.0001 | -8953.2  (-10744.9 to -7161.5) | <0.0001 | -12787.1  (-15981.7 to -9592.5) | <0.0001 |

Descriptive statistics are shown as mean difference (95% confidence interval).

**Table E6.** Effect (positive *vs* negative) of MCC950 on nigericin- and LPS+Nigericin-induced IL-1β secretion from peripheral blood mononuclear cells stratified based on obesity status

|  | **Non-obese** | | | | **Obese** | | | |
| --- | --- | --- | --- | --- | --- | --- | --- | --- |
|  | **Healthy/Non-severe** | | **Severe** | | **Healthy/Non-severe** | | **Severe** | |
|  | **Mean diff (95% CI)** | ***P* Value** | **Mean diff (95% CI)** | ***P* Value** | **Mean diff (95% CI)** | ***P* Value** | **Mean diff (95% CI)** | ***P* Value** |
| Media (2 h) + Nigericin (1 h) | -77.5  (-116.0 to  -39.0) | 0.0001 | -271.9  (-441.4 to  -102.5) | 0.0022 | -139.2  (-194.8 to  -83.5) | <0.0001 | -194.6  (-255.9 to  -133.2) | <0.0001 |
| Media (4 h) + Nigericin (1 h) | -50.3  (-79.1 to  -21.5) | 0.0009 | -166.6  (-265.8 to  -67.4) | 0.0014 | -80.9  (-117.1 to  -44.7) | <0.0001 | -112.8  (-155.2 to  -70.5) | <0.0001 |
| LPS (2 h) + Nigericin (1 h) | -3332.3  (-3917.0 to  -2747.6) | <0.0001 | -5729.6  (-7085.1 to  -4374.1) | <0.0001 | -2983.9  (-3360.9 to  -2606.8) | <0.0001 | -4177.1  (-5232.9 to  -3121.4) | <0.0001 |
| LPS (4 h) + Nigericin (1 h) | -8800.1  (-10217.1 to -7383.1) | <0.0001 | -15318.1  (-19574.4 to  -11061.9) | <0.0001 | -7450.1  (-8376.0 to  -6524.3) | <0.0001 | -10083.2  (-12046.7 to  -8119.7) | <0.0001 |

Descriptive statistics are shown as mean difference (95% confidence interval).

**Table E7.** Nigericin- and LPS+Nigericin-induced IL-1β secretion from peripheral blood mononuclear cells from asthmatic subjects (severe and non-severe subjects combined) stratified based on eosinophilic or non-eosinophilic asthma status.

| **Treatment** | ***P* Value** | **LSMean diff**  **(Eosinophilic vs Non-Eosinophilic)** |
| --- | --- | --- |
| Media (2 h) | 0.3243 | 3.3 (-3.2 to 9.8) |
| Media (4 h) | 0.5999 | -0.4 (-1.9 to 1.1) |
| Media (2 h) + Nigericin (1 h) | 0.1554 | 64.0 (-24.0 to 151.9) |
| Media (4 h) + Nigericin (1 h) | 0.1754 | 38.0 (-16.8 to 92.8) |
| LPS (2 h) | 0.1721 | 33.6 (-14.5 to 81.8) |
| LPS (4 h) | 0.6680 | 41.1 (-146.5 to 228.8) |
| LPS (2 h) + Nigericin (1 h) | 0.4283 | -440.4 (-1528.0 to 647.2) |
| LPS (4 h) + Nigericin (1 h) | 0.1017 | -2326.3 (-5100.8 to 448.1) |

Descriptive statistics are shown as Least Squares Mean difference.

**Figure E1.** *Ex vivo* peripheral blood mononuclear cell (PBMC) stimulations with LPS (lipopolysaccharide) for 2 or 4 hours, nigericin for 1 hour, and treatment with the highly specific NLRP3 inflammasome inhibitor, MCC950 (1 hour). All controls were sham stimulated with media (sham for LPS) or PBS (sham for nigericin/MCC950).
